# Dietary *Debaryomyces hansenii* promotes skin and skin mucus defensive capacities in a marine fish model

**DOI:** 10.3389/fimmu.2023.1247199

**Published:** 2023-08-30

**Authors:** Ignasi Sanahuja, Laura Fernandez-Alacid, Silvia Torrecillas, Alberto Ruiz, Eva Vallejos-Vidal, Joana P. Firmino, Felipe E. Reyes-Lopez, Lluis Tort, Dariel Tovar-Ramirez, Antoni Ibarz, Enric Gisbert

**Affiliations:** ^1^ Aquaculture Program, Institute of Agrifood Research and Technology (IRTA), La Ràpita, Spain; ^2^ Department of Cell Biology, Physiology, and Immunology, University of Barcelona, Barcelona, Spain; ^3^ Núcleo de Investigaciones Aplicadas en Ciencias Veterinarias y Agronómicas, Facultad de Medicina Veterinaria y Agronomía, Universidad de Las Américas, Santiago, Chile; ^4^ Centro de Biotecnología Acuícola, Universidad de Santiago de Chile, Santiago, Chile; ^5^ Department of Cell Biology, Physiology, and Immunology, Universitat Autonoma de Barcelona, Barcelona, Spain; ^6^ Centro de Investigaciones Biológicas del Noroeste SC, CIBNOR, La Paz, Mexico

**Keywords:** *Debaryomyces hansenii*, skin defense, skin mucus defense, skin mucus associated biomarkers, transcriptomics, yeast probiotic

## Abstract

The present study explores the effects of two supplementation levels of *Debaryomyces hansenii* (1.1% and 2.2%) as a probiotic in a reference low fish meal-based diet on the skin mucosal tissue in *Sparus aurata*. This study includes the evaluation of fish performance coupled with a holistic study of the skin mucosa: i) a transcriptomic study of the skin tissue, and ii) the evaluation of its secreted mucus both in terms of skin mucosal-associated biomarkers and its defensive capacity by means of co-culture analysis with two pathogenic bacteria. Results showed that after 70 days of diet administration, fish fed the diet supplemented with *D. hansenii* at 1.1% presented increased somatic growth and a better feed conversion ratio, compared to fish fed the control diet. In contrast, fish fed the diet including 2.2% of the probiotic presented intermediate values. Regarding gene regulation, the probiotic administration at 1.1% resulted in 712 differentially expressed genes (DEGs), among which 53.4% and 46.6% were up- and down-regulated, respectively. In particular, *D. hansenii* modulated some skin biological processes related to immunity and metabolism. Specifically, *D. hansenii* administration induced a strong modulation of some immune biological-related processes (61 DEGs), mainly involved in B- and T-cell regulatory pathways. Furthermore, dietary *D. hansenii* promoted the skin barrier function by the upregulation of anchoring junction genes (23 DEGs), which reinforces the physical defense against potential skin damage. In contrast, the skin showed modulated genes related to extracellular exosome and membrane organization (50 DEGs). This modulated functioning is of great interest, particularly in relation to the increased skin mucus defensive capacity observed in the bacterial co-culture *in vitro* trials, which could be related to the increased modulation and exudation of the innate immune components from the skin cells into the mucus. In summary, the modulation of innate immune parameters coupled with increased skin barrier function and cell trafficking potentiates the skin’s physical barrier and mucus defensive capacity, while maintaining the skin mucosa’s homeostatic immune and metabolic status. These findings confirmed the advantages of *D. hansenii* supplementation in low fish meal-based diets, demonstrating the probiotic benefits on cultured marine species.

## Graphical Abstract

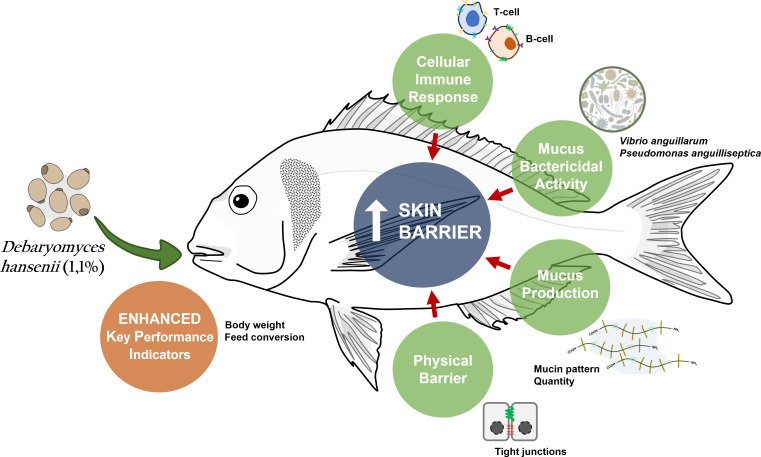

## 1 Introduction

The primary function of the epidermis is to separate the animals’ internal medium from the external environment, and this function has been modulated through species by physical and chemical adaptations (1). In fish, mucosal structures are considered mucosal-associated lymphoid tissues (MALT), which means that these specialized structures are capable of sensing and responding to environmental and biotic challenges (2). For this purpose, the mucosal immune system in fish has acquired throughout evolution a wide variety of interconnected cells with innate and adaptive components (3). Moreover, mucosae are dynamic and semipermeable barriers with several physiological capacities, among which are osmoregulation, respiration, nutrition, or locomotion (4–6). These characteristics could be modulated either by: (i) the improvement of husbandry and farming practices or (ii) providing a balanced diet. On this basis, adequate nutrition could avoid health deficiencies, meanwhile maintaining fish performance and protecting animals from biotic and abiotic stressors (7). In addition, nutritional strategies could be used to enhance fish health while promoting a sustainable practice by reducing the dependence on traditional marine raw materials (8, 9). However, balancing nutrients in aquafeeds with alternative resources to the fish-derived products is complex and usually a bottleneck to fish development and health (10).

One step further in the search for quality nutrition is supplementation to enhance certain aspects of fish biology or to counteract the deficiencies of sustainable non-animal-based aquafeeds. Among other supplements, prebiotics and probiotics confer benefits to the host by enhancing the gastrointestinal milieu, optimizing nutrient digestion and absorption, improving growth performance, and modulating the fish’s immune system (11, 12). These products could also possess mechanisms to modulate microbial communities, either by maintaining native microbiota or by introducing beneficial organisms that establish a synergistic symbiosis relevant to the host (13). One of these feed additives that globally encompass all the above-mentioned benefits is yeasts (14). Yeasts are single-celled organisms that contain wall-related bioactive compounds, which function as stimulants, favoring growth and the immune system and contributing to the overall animal condition and health (15). *Debaryomyces hansenii*, a yeast that belongs to the natural microbiota of carnivorous fish, offers different benefits in terms of health and performance, which in the aquaculture industry is of great interest. *D. hansenii* has been reported to enhance larval survival and development in several marine fish species (16–18), whereas in juveniles, it is reputed for promoting the immune system (17, 19–21). Several authors have attributed the beneficial effects of yeast to their β-glucan and polyamine wall-related contents (22, 23); however, its potential benefits and mode of action at the level of mucosal-associated lymphoid tissues have been poorly studied.

Focusing on MALT, available literature on *D. hansenii* primarily addresses its effects on gut-associated lymphoid tissue (GALT). Research has found that *D. hansenii* improves intestinal function and immunity, meanwhile modulating gut microbiota (19, 20, 23–25), which are effects that may be associated with its positive impacts on fish growth and performance (21). However, little is known about the effects of a *D. hansenii* dietary supplementation on other MALT, and especially the effects on the principal mucosa barrier against the environment, the skin. The skin and its exuded mucus present a similar multifactorial behavior against the environment (26), and due to their direct contact with the aquatic environment, play a principal role in protection (27). An example of this involvement is the production of a specialized mucus layer with a wide range of physical and biochemical defensive components (28, 29), which may be modulated through several factors, including dietary supplementation (30–32).

The present study aimed to determine the effects of a low fish meal-based diet (7%), supplemented with *D. hansenii*, in the skin mucosa of gilthead seabream (*Sparus aurata*). To provide insight into the effects of this probiotic on gilthead seabream skin, we combined a skin transcriptomic analysis, with the modification of the skin mucus-associated biomarkers (SMABs), in addition to the study of its antibacterial capacity through a co-culture with two pathogenic bacteria for this fish species.

## 2 Materials and methods

### 2.1 Diet composition

Three experimental diets were designed to evaluate the effects of *D. hansenii* (CBS 8339) on fish performance and feed utilization and evaluate the effects of its supplementation on the skin through transcriptomic analysis. A 7% fish meal-based basal diet was formulated and considered a control diet (**Table 1**). Subsequently, the control diet was used as a basal diet to create both experimental yeast diets, which only differed on *D. hansenii* content proportion (Yeast_1.1%_ diet with 1.1%, 17.2 x 10^5^ cfu; and Yeast_2.2%_ diet 2.2%, 35.7 x 10^5^ cfu) as shown in **Table 1**. These percentages have been determined in previous studies (17–19, 21). Diets were isoproteic (48.4%), isolipidic (17.2%), and isoenergetic (21.7 MJ/Kg feed). Each of the diets (pellet size: 2mm) was manufactured by SPAROS Lda (Portugal) under the conditions detailed by Gisbert et al. (33) and stored at 4°C throughout the experiment to prevent feed deterioration. Diet manufacture conditions, packaging, and storage did not compromise the viability of the probiotic (18, 20, 21). *D. hansenii* was provided by CIBNOR (La Paz, Mexico) and cultured as described by Tovar-Ramírez et al. (18).

**Table 1:** Ingredients and proximal composition of the experimental diets.

	DIETS		
	Control	Yeast_1.1%_	Yeast_2.2%_
*Feed basis Ingredients (%)*			
Fishmeal^a^	7.0	7.0	7.0
Soy protein concentrate^b^	21.0	21.0	21.0
Pea protein concentrate	12.0	12.0	12.0
Wheat gluten	12.0	12.0	12.0
Corn gluten	12.0	12.0	12.0
Soybean meal 48	5.0	5.0	5.0
Wheat meal	10.4	10.4	10.4
Fish oil^c^	15.0	15.0	15.0
Vit. and Min. Premix (PV01)	1.0	1.0	1.0
Soy lecithin – Powder	1.0	1.0	1.0
Binder (guar gum)	1.0	1.0	1.0
Monocalcium phosphate	2.0	2.0	2.0
L-Lysine	0.3	0.3	0.3
L-Tryptophan	0.1	0.1	0.1
DL-Methionine	0.2	0.2	0.2
*Supplementation (%)*			
Yeast (*Debaryomyces hansenii*), %	–	1.1	2.2
*Feed basis proximate composition*			
Crude protein, % feed	48.4	48.4	48.4
Crude fat, % feed	17.2	17.2	17.2
Fiber, % feed	1.53	1.53	1.53
Ash, % feed	5.89	5.89	5.89
Gross energy, MJ/Kg feed	21.7	21.7	21.7

a Fishmeal LT70, Norvik 70, Sopropêche, France.

b SOYCOMIL^®^, ADM Animal Nutrition, Quincy, USA.

c SAVINOR UTS, Trofa, Portugal.

d Vitamin and mineral premix: PREMIX Sparos Lda, Portugal: Vitamins (IU or mg/kg diet): DL-alpha tocopherol acetate, 100 mg; sodium menadione bisulfate, 25 mg; retinyl acetate, 20000 IU; DL-cholecalciferol, 2000 IU; thiamin, 30 mg; riboflavin, 30 mg; pyridoxine, 20 mg; cyanocobalamin, 0.1mg; n-icotinic acid, 200 mg; folic acid, 15 mg; ascorbic acid, 500 mg; inositol, 500 mg; biotin, 3 mg; calcium pantothenate, 100mg; choline chloride, 1,000m g, betaine, 500 mg. Minerals (g or mg/kg diet): copper sulfate, 9 mg; ferric sulfate, 6 mg; potassium iodide, 0.5 mg; manganese oxide, 9.6 mg; sodium selenite, 0.01 mg; zinc sulfate,7.5 mg; sodium chloride, 400 mg; excipient wheat middlings.

### 2.2 Fish and experimental model design

A total of 500 *S. aurata* juveniles (BW = 14.5 ± 1.2 g) were obtained from a commercial farm (PISCIMAR SL; AVRAMAR, Burriana, Spain) and transported and acclimated to IRTA-La Ràpita research facilities (La Ràpita, Spain). During acclimatization, fish were fed a commercial diet (Optibream, Skretting) and maintained in a 2,000 L tank connected to IRTAmar^®^ recirculating system under natural conditions and constant temperature (23.0 ± 1.0°C).

Prior to the experiment, 300 fish were slightly anesthetized (50 mg/L tricaine methanesulfonate, MS-222, Sigma-Aldrich, Madrid, Spain) and randomly distributed in four replicate tanks per diet (N = 25 fish per tank). The experiment lasted for 70 days, and diets were distributed in four meals at 08:00, 11:00, 13:00, and 16:00 h (the corresponding feed ratio was distributed during 1 h), and uneaten pellets were collected and weighed to calculate daily feed ingesta values. The trial was run under natural photoperiod (14h light/8h dark), salinity (35-36 ‰), constant temperature (23.3 ± 1.3°C), dissolved oxygen (5.7 ± 0.2 mg/L) (OXI330, Crison Instruments, Spain), and pH (8.2 ± 0.1) (pHmeter 507, Crison Instruments). Ammonia and nitrite levels (0.13 ± 0.1 mg /L and 0.18 ± 0.1 mg /L, respectively) were continuously controlled using HACH DR 900 Colorimeter (Hach Company, Spain).

Growth performance and feed utilization indicators were calculated using the following formulae:

- Weight gain rate (WGR) % = 100 x [(BWf – BWi)/BWi]; where BWf and BWi are the final and initial mean BW of fish.

- Specific growth rate (SGR) % BW/day = 100 x [(ln BWf – ln BWi)/days]

- Survival rate (SR) % = 100 x (Final number of fish/Initial number of fish).

- Feed conversion ratio (FCR) = Feed intake (g)/Increase in fish biomass (g).

At the end of the experiment, all fish were anesthetized (100 mg/L tricaine methanesulfonate, MS222) and measured individually for the calculation of key performance indicators as previously described and to sample skin mucus following the method described by Fernandez-Alacid et al. (34). According to the results obtained from the growth and skin mucus metabolites, only control and Yeast_1.1%_ diets were considered for the skin transcriptomics and skin mucus defensive capacity analyses.

In addition, three randomly selected fish per replicate tank (N = 12) were euthanized (300 mg/L MS222) for transcriptional analysis purposes. A ca. 1 cm^2^ of skin section from the mid-region of the fish body (over the lateral line, right side of each fish) was dissected and the muscle tissue attached to it was discarded. Samples were incubated overnight (4°C) in RNAlater™ and stored at -80°C for further RNA extraction.

### 2.3 Skin mucus analyses

#### 2.3.1 Skin mucus collection and associated biomarkers

At final sampling, 36 animals were randomly selected (12 fish for each dietary condition; three to four fish per tank) and their skin mucus was collected following the method described by Fernández-Alacid et al. (34). Glucose and lactate concentrations on skin mucus were determined by an enzymatic colorimetric test (LO-POD glucose, SPINREACT^®^, St. Esteve de Bas, Spain) and (LO-POD lactate, SPINREACT^®^, Barcelona, Spain), respectively, following the manufacturer’s instructions, but with slight modifications for fish skin mucus as described by Fernandez-Alacid et al. (34). Glucose and lactate values were expressed as μg/mL of skin mucus. Protein concentration (mg/mL of skin mucus) was determined using the Bradford assay (35), using bovine serum albumin as standard (BSA; Sigma Aldrich, Madrid, Spain). Cortisol levels were measured using an ELISA kit (IBL International, Tecan Group, Switzerland) following the manufacturer’s instructions for saliva determinations but with slight modifications for fish skin mucus (34). Cortisol values were expressed as ng/mL of skin mucus. The Ferric Antioxidant Power (FRAP) was measured by means of an enzymatic colorimetric test (Invitrogen, Thermo Fisher Scientific, Spain), following the manufacturer’s instructions for plasma, with minor modifications; values were expressed as µmol/mL of mucus. Mucus ratios referring to protein (glucose/protein, lactate/protein, cortisol/protein, and FRAP/protein) were calculated in order to avoid the dilution derived from mucus sampling. The glucose/lactate ratio was also calculated as a metabolic response indicator (34).

#### 2.3.2 Bacterial growth assessment in skin mucus

To study the skin mucus antibacterial capacity, three different bacteria were tested: two pathogenic bacteria, *Vibrio anguillarum* (CECT522T) and *Pseudomonas anguilliseptica* (CECT899T), and a non-pathogenic bacterium, *Escherichia coli* (DSMZ423). The pathogenic *V. anguillarum* and *P. anguilliseptica* strains were grown in Marine Broth culture media (MB, Difco Laboratories, Detroit) and the non-pathogenic *E. coli* strain was grown in Tryptic Soy Broth culture media (TSB, Conda, Spain). The skin mucus antibacterial capacity (from control and Yeast_1.1%_ diets) on three strains’ viability was evaluated by monitoring the absorbance of co-culture grown at 400nm (per triplicate) for 14 hours in 96-well plates, as described by Fernandez-Alacid et al. (36).

### 2.4 Skin transcriptional analysis

#### 2.4.1 RNA isolation and quality control

Total RNA was extracted from the skin of 12 randomly selected fish per feeding treatment using the RNeasy^®^ Mini Kit (Qiagen, Germany). Total RNA was eluted in a final volume of 35 µL nuclease-free and processed with DNAse (DNA-free™ DNA removal Kit; Invitrogen, Lithuania). Total RNA concentration and purity were measured using a Nanodrop-2000^®^ spectrophotometer (Thermo Scientific, USA) and stored at -80°C for further analysis. Prior to microarray hybridization, the RNA samples were diluted to a concentration of 133.33 ng/µL and tested for RNA integrity (Agilent 2100 Bioanalyzer; Agilent Technologies, Spain). The samples were selected according to the criteria for RIN value > 8.5. Three different pools for each diet regimen were selected (N = four fish per pool).

#### 2.4.2 Microarray hybridization and analysis

Skin transcriptional analysis from both experimental groups was carried out using the Aquagenomics *Sparus aurata* oligonucleotide microarray v2.0 (4 × 44 K) (SAQ) platform. Detailed data and transcriptomic raw data are in the National Center for Biotechnology Information (NCBI)’s Gene Expression Omnibus (GEO) public repository under accession numbers GPL13442 and GSE162504, respectively. Sampling labeling, hybridization, washing, and scanning were performed as described in (37). Briefly, a one-color RNA labeling (Agilent One-Color RNA Spike-In kit; Agilent Technologies, USA) was used. RNA from each pool (200 ng) was reverse-transcribed using spike-in. Total RNA was used as a template for Cyanine-3 (Cy3)-labeled cRNA synthesis and amplified using a Quick Amp Labeling kit (Agilent Technologies). cRNA samples were purified using an RNeasy^®^ micro kit (Qiagen). Dye incorporation and cRNA yield were checked (NanoDrop ND-2000^®^ spectrophotometer). Then, Cy3-labeled cRNA (1.5 mg) with >6.0 pmol Cy3/mg specific activity was then fragmented at 60°C for 30 min and hybridized at 65°C with the array in the presence of hybridization buffer (Gene expression hybridization kit, Agilent Technologies) for 17 h. For washes, microarrays were incubated with Gene expression wash buffers, and stabilization and drying solutions were according to the manufacturer’s instructions (Agilent Technologies). Microarray slides were scanned (Agilent G2505B Microarray Scanner System), and spot intensities and other quality control features were extracted (Agilent Feature Extraction software version 10.4.0.0).

The Search Tool for the Retrieval of Interacting Genes (STRING) public repository version 11.0 (https://string-db.org) was used to construct skin transcripteractome comparing fish fed with the control and Yeast_1.1%_ diets. Protein-Protein interaction (PPI) Networks Functional Enrichment Analysis for all the differentially expressed genes (DEGs) was conducted with a medium-confidence interaction score (0.4) using *Homo sapiens* as the model organism (37, 38). Gene Ontology (GO) and Kyoto Encyclopedia of Genes and Genomes (KEGG) enrichment analysis of all the DEGs were also evaluated through STRING (*p* < 0.05). A human orthology identification based on gene/protein name was accessed through the Genecards (www.genecards.org) (39) and Uniprot (www.uniprot.org) databases to confirm the match of gene acronyms between both *H. sapiens* and *S. aurata*. Additionally, protein-protein BLAST (BLASTp) was run (E < 10^−7^; query cover > 95%). The cluster aggrupation of nodes classified according to their biological processes was represented with the application ClueGO (v2.5.9) and CluePedia (v1.5.9) using Cytoscape (v3.9.1) software.

### 2.5 Statistical analysis

Equality of variances and normality were checked using Levene’s test. Differences among the key performance parameters and SMABs were performed using a one-way ANOVA test, followed by *post-hoc* Bonferroni’s test (if equal variances were assumed) or Dunnett’s test (if variances among groups were unbalanced) to test the effects of yeast supplementation levels (*p* < 0.05). In addition, curvilinear estimation of regression analysis was performed to a deep comprehension of KPI and SMAB differences (*p* < 0.05). Student’s *t*-test, assuming data homoscedasticity, was used to compare bacterial growth differences among the Yeast_1.1%_ diet and control diet (*p* < 0.05). Data on KPI and SMABs were expressed as mean values ± standard deviations using SPSS Statistics for Windows (v.25; IBM Corp., Armonk, NY, USA), and bacterial growth values were expressed as mean ± standard error, with GraphPad PRISM 9.

## 3 Results

### 3.1 Key performance indicators

Results on fish growth performance and key performance indicators are represented and summarized in **Table 2**. Briefly, fish fed the Yeast_1.1%_ diet performed better than the rest of the dietary groups. Values of WGR, SGR, and FCR were improved in fish fed the Yeast_1.1%_ diet compared to those from both control and Yeast_2.2%_ diets. Furthermore, the feed conversion ratio (FCR) value was lower (*p* < 0.05) in fish fed the Yeast_1.1%_ diet when compared to fish fed the reference diet, whereas fish fed the Yeast_2.2%_ diet showed an intermediate value. However, no significant differences in feed intake (FI) were found among the experimental groups (**Table 2**; *p* > 0.05).

**Table 2:** Growth and feed performance indicators in gilthead sea bream (*Sparus aurata*) fed the experimental diets containing *Debaryomyces hansenii* (1.1%, and 2.2%) or devoid of the probiotic (Control diet).

	Control		Yeast_1.1%_		Yeast_2.2%_		Regression		
							Model	R^2^	Sig.
Final body weight (g)	84.4 ± 2.92	A	95.6 ± 4.64	B	85.6 ± 1.19	A	Qu	0.761	0.002
Weight gain rate (%)	482.6 ± 20.1	A	559.4 ± 32.0	B	490.0 ± 8.2	A	Qu	0.762	0.002
Specific growth rate (% BW/day)	2.52 ± 0.05	A	2.69 ± 0.07	B	2.54 ± 0.02	A	Qu	0.764	0.002
Survival rate (%)	92.0 ± 3.3	–	93.0 ± 2.0	–	93.0 ± 2.0	–	Ns	–	–
Feed intake (g)	86.9 ± 3.77	–	84.3 ± 6.65	–	80.4 ± 5.22	–	Ns	–	–
Feed conversion ratio	1.26 ± 0.07	A	1.06 ± 0.05	B	1.14 ± 0.08	AB	Qu	0.675	0.006

Data are shown as the mean ± standard deviation (SD). The letters denote statistically significant differences among groups (ANOVA, p < 0.05; n = 4). Regression indicates the best curve fit estimation model. Sig, Significance; Ns, No significant; Qu, Quadratic response.

### 3.2 Skin mucus analyses

#### 3.2.1 Skin mucus associated biomarkers

Values of SMABs and their ratios, as well as the FRAP from skin mucus samples, are summarized in **Table 3**. No differences between the control and Yeast_1.1%_ dietary groups were observed in terms of skin mucus biomarkers nor when they were normalized by protein values (*p* > 0.05). However, higher yeast supplementation (2.2% yeast diet) increased glucose levels in fish skin mucus, which was maintained even with protein normalization (*p* < 0.05).

**Table 3:** Skin mucus-associated biomarkers (SMABs) of gilthead seabream (*Sparus aurata*) fed an experimental diet supplemented with 1.1% (Yeast_1.1%_) and 2.2% (Yeast_2.2%_) of *D. hansenii*, and the control diet devoid of the feed additive.

	Control		Yeast_1.1%_		Yeast_2.2%_		Regression		
							Model	R^2^	Sig.
*SMABs*									
Glucose (µg/mL)	8.93 ± 2.04	A	10.15 ± 6.23	AB	23.02 ± 13.66	B	Ln	0.317	0.015
Lactate (µg/mL)	12.18 ± 6.24	–	20.05 ± 19.17	–	32.17 ± 28.39	–	Ns	–	–
Protein (mg/mL)	8.97 ± 3.60	–	10.73 ± 6.12	–	10.07 ± 4.99	–	Ns	–	–
Cortisol (ng/mL)	0.60 ± 0.22	–	0.62 ± 0.26	–	0.66 ± 0.26	–	Ns	–	–
FRAP (µmol/ml)	1588.2 ± 455.3	–	1549.4 ± 448.9	–	–	–	Ns	–	–
*SMABs ratios*									
Glucose/Pr (µg/mg)	1.11 ± 0.37	A	0.94 ± 0.23	A	1.83 ± 0.47	B	Qu	0.570	0.012
Lactate/Pr (µg/mg)	1.62 ± 0.76	–	1.56 ± 0.87	–	2.68 ± 1.37	–	Ns	–	–
Gluc/Lac (mg/mg)	0.69 ± 0.34	–	0.77 ± 0.38	–	0.71 ± 0.23	–	Ns	–	–
Cortisol/Pr (ng/g)	109.4 ± 65.7	–	94.4 ± 68.3	–	92.1 ± 40.5	–	Ns	–	–
FRAP/Pr (µmol/mg)	184.8 ± 65.7	–	138.5 ± 47.9	–	–	–	Ns	–	–

Data are shown as the mean ± standard deviation (SD). The letters denote statistically significant differences among groups (one-way ANOVA, p < 0.05; n = 12). Regression indicates the best curve fit estimation model. Sig, Significance; Ln, linear response; Ns, No significant; Qu, Quadratic response.

#### 3.2.2 Skin mucus antibacterial capacity

We evaluated the skin mucus’s defensive capacity to know whether the diet supplemented with yeast implies a functional protective mechanism against pathogenic bacterial growth on skin mucus. Taking into account results from KPI related to growth and feed utilization, only skin mucus samples from fish fed the control and the Yeast_1.1%_ diets were analyzed.

**Figure 1:**
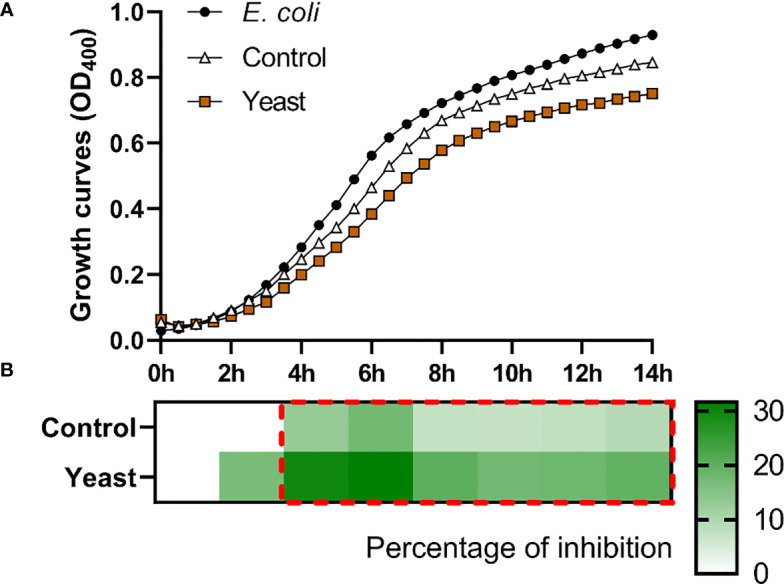
Co-culture growth of *E. coli* on the skin mucus of gilthead seabream (*Sparus aurata*) fed experimental low fishmeal diets containing *Debaryomyces hansenii* (1.1%, 17.2 x 10^5^ cfu; Yeast diet) or devoid of the probiotic (Control diet). Data from growth curves **(A)** correspond to the mean ± SEM of triplicate samples. Black circles correspond to *E. coli* growth in a medium devoid of mucus; white triangles correspond to *E. coli* growth in the mucus of fish fed the Control diet; brown squares correspond to *E. coli* growth in the mucus of fish fed the Yeast_1.1%_ diet. The heat map shows the percentage of inhibition **(B)** of both diets in a red (negative) to green (positive) gradient. The dashed red line square indicates significant differences in bacterial growth between dietary groups (Student’s *t-*test, *p* < 0.05).

**Figure 2:**
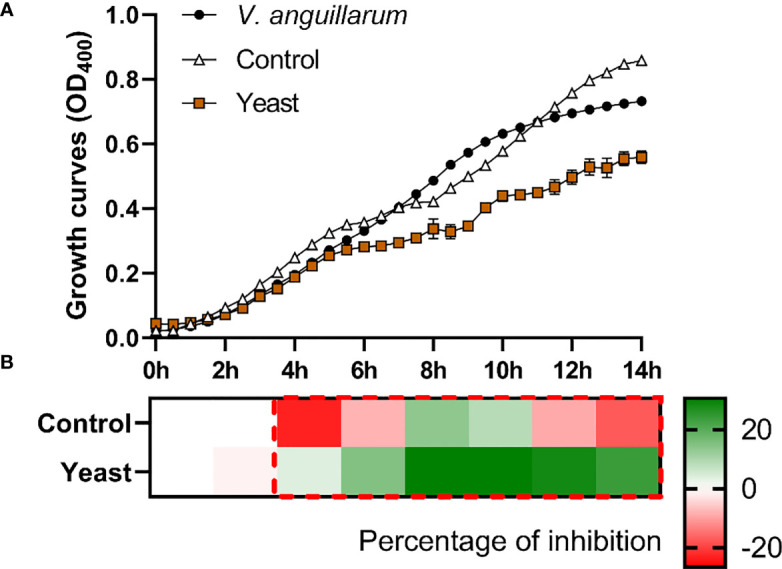
Co-culture growth of *V. anguillarum* on the skin mucus of gilthead seabream (*Sparus aurata*) fed experimental low fishmeal diets containing *Debaryomyces hansenii* (1.1%, 17.2 x 10^5^ cfu; Yeast diet) or devoid of the probiotic (Control diet). Data from growth curves **(A)** correspond to the mean ± SEM of triplicate samples. Black circles correspond to *V. anguillarum* growth in a medium devoid of mucus; white triangles correspond to *V. anguillarum* growth in the mucus of fish fed the Control diet; and brown squares correspond to *V. anguillarum* growth in the mucus of fish fed the Yeast_1.1%_ diet. The heat map shows the percentage of inhibition **(B)** of both diets in a red (negative) to green (positive) gradient. The dashed red line square indicates significant differences in bacterial growth between dietary groups (Student’s *t*-test, *p* < 0.05).

**Figure 3:**
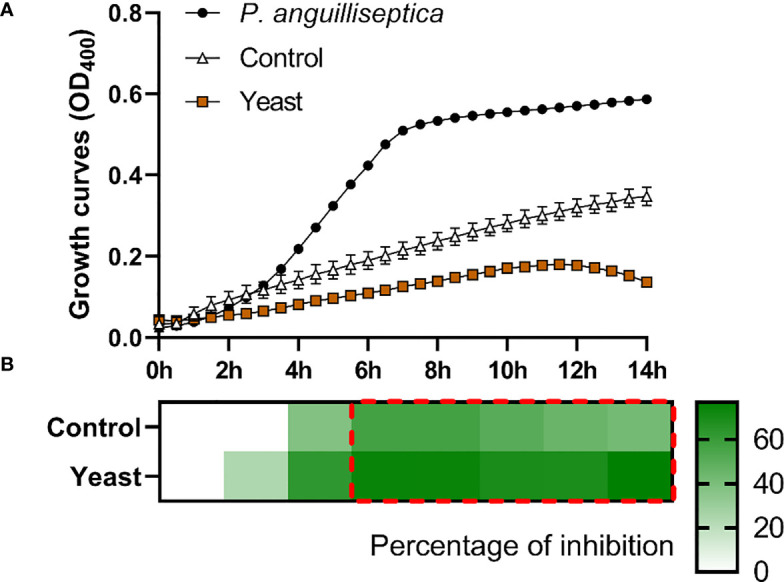
*P. anguilliseptica* co-culture growth on the skin mucus of gilthead seabream (*Sparus aurata*) fed experimental low fishmeal diets containing *Debaryomyces hansenii* (1.1%, 17.2 x 10^5^ cfu; Yeast diet) or devoid of the probiotic (Control diet). Data from growth curves **(A)** correspond to the mean ± SEM of triplicate samples. Black circles correspond to *P. anguilliseptica* growth in a medium devoid of mucus; white triangles correspond to *P. anguilliseptica* growth in the mucus of fish fed the Control diet; and brown squares correspond to *P. anguilliseptica* growth in the mucus of fish fed the Yeast_1.1%_ diet. The heat map shows the percentage of inhibition **(B)** of both diets in a red (negative) to green (positive) gradient. The dashed red line square indicates significant differences in bacterial growth between dietary groups (Student’s *t*-test, *p* < 0.05).

The *S. aurata* skin mucus from both nutritional groups (Control and Yeast_1.1%_) showed a decrease in *E. coli* (**Figure 1**), *V. anguillarum* (**Figure 2**), and *P. anguilliseptica* (**Figure 3**) growth during all the co-culture periods. When cultured with the skin mucus from fish fed the Yeast_1.1%_ diet, a reduction in the growth of the non-pathogenic bacteria *E. coli* was observed (*p* < 0.05; **Figure 1A**). Growth decrease was recorded between 4 and 14 h of bacterial culture. The most accentuated decrease in growth values compared with fish fed the control diet was found in the logarithmic phase (approximately 15% between 4h and 6h, 12% at 8h, and 10% until 14h) (**Figure 1B**). Regarding *V. anguillarum*, a decrease in bacterial growth was observed in the co-culture with the skin mucus from fish fed the supplemented diet (**Figure 2A**), being the most remarkable growth decrease between 8h-14h. In addition, the mucus of the fish fed with the Yeast_1.1%_ diet had a substantial growth inhibiting capacity, being 20-30% greater than fish fed the control diet (**Figure 2B**). However, the most accentuated growth decline was observed in *P. anguilliseptica* (**Figure 3**), registering a maximum decrease in bacterial growth between 6h and 14h (percentage of inhibition over 20% compared with the control diet, *p* < 0.05) associated with the skin mucus of fish fed the yeast-supplemented diet.

### 3.3 Skin transcriptomics

#### 3.3.1 Transcriptomic profile

**Figure 4:**
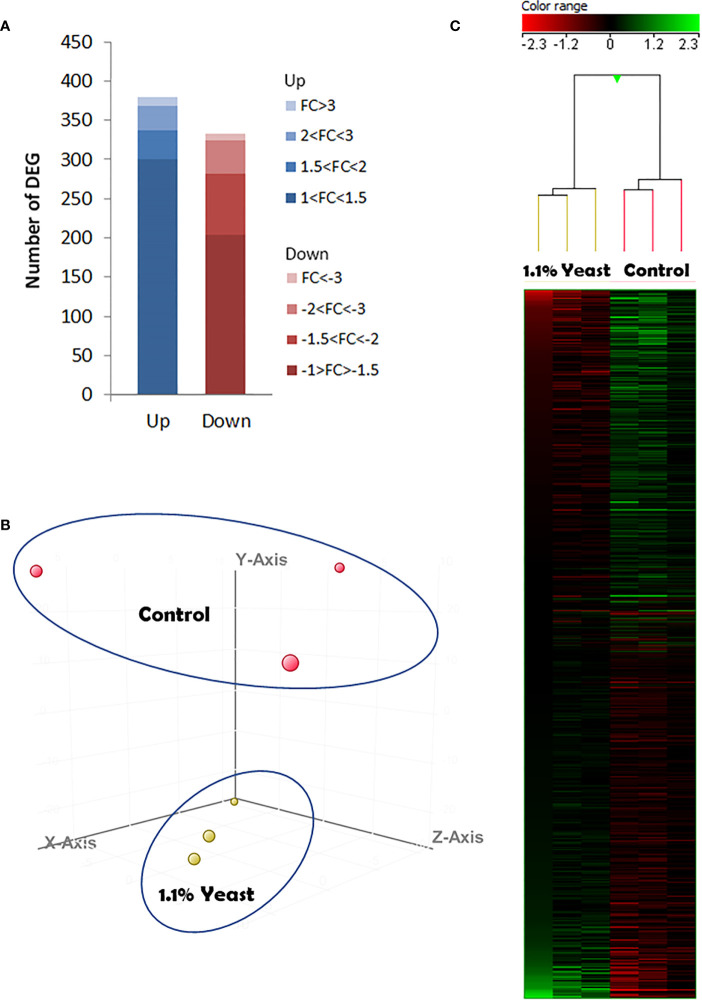
Skin microarrays-based transcriptomic analysis for the skin of gilthead seabream (Sparus aurata) fed experimental low fishmeal diets containing Debaryomyces hansenii (1.1%, 17.2 x 10^5^ cfu; Yeast_1.1%_ diet) or devoid of the probiotic (Control diet). **(A)** The number of total differentially expressed genes (DEGs). The blue (up-regulated) and red (down-regulated) color schemes indicate the gene modulation according to its magnitude interval (fold-change). **(B)** 3D Principal component analysis (PCA). Control (red spheres) and D. hansenii groups (yellow spheres) are represented (each array included in the study is represented by one sphere). **(C)** Hierarchical clustering heatmap representing the 712 DEGs. The results of each microarray analyzed for the control and D. hansenii group are shown. The green (up-regulation) and red (down-regulation) color schemes indicate the gene normalized intensity values according to its magnitude interval.

In order to determine the effects of dietary supplementation of the yeast *D. hansenii* upon the *S. aurata* skin transcriptome, we conducted a microarray-based transcriptomic analysis. As results from fish condition neither indicated statistical differences between the Control and Yeast_2.2%_ groups nor linear regression determined a dose-response effect, skin transcriptomics was only analyzed from fish fed the Yeast_1.1%_ diet that showed the best results in terms of BW_f_ and FCR values. A total of 712 differentially expressed genes (DEGs) were found in the skin of *S. aurata* fed the Yeast_1.1%_ diet (**Figure 4** and **Supplementary Table 1**). The DEGs were classified as up-regulated (UR-DEGs; N = 380) or down-regulated (DR-DEGs; N = 332) and ordered by their fold-change (FC) as shown in **Figure 4A**. The FC values obtained showed similar modulations at high-expressions ([FC ≥ 2], UR-DEGs = 11.3% and DR-DEGs = 15.7%), but at mid- and low-expressions the modulation percentages were remarkable ([1.5 ≤ FC < 2], UR-DEGs = 9.7% and DR-DEGs = 23.2%; [1 ≤ FC < 1.5], UR-DEGs = 79% and DR-DEGs = 61.1%). When comparing both inter- and intra-specific profiles from both experimental groups using a hierarchical clustering heatmap, a common aggregation among pool samples and an inverted response based on dietary administration was observed (**Figure 4C**). These results were supported by the PCA analysis showing differential profiles among dietary treatments in a Euclidean 3D space (**Figure 4B**).

**Figure 5:**
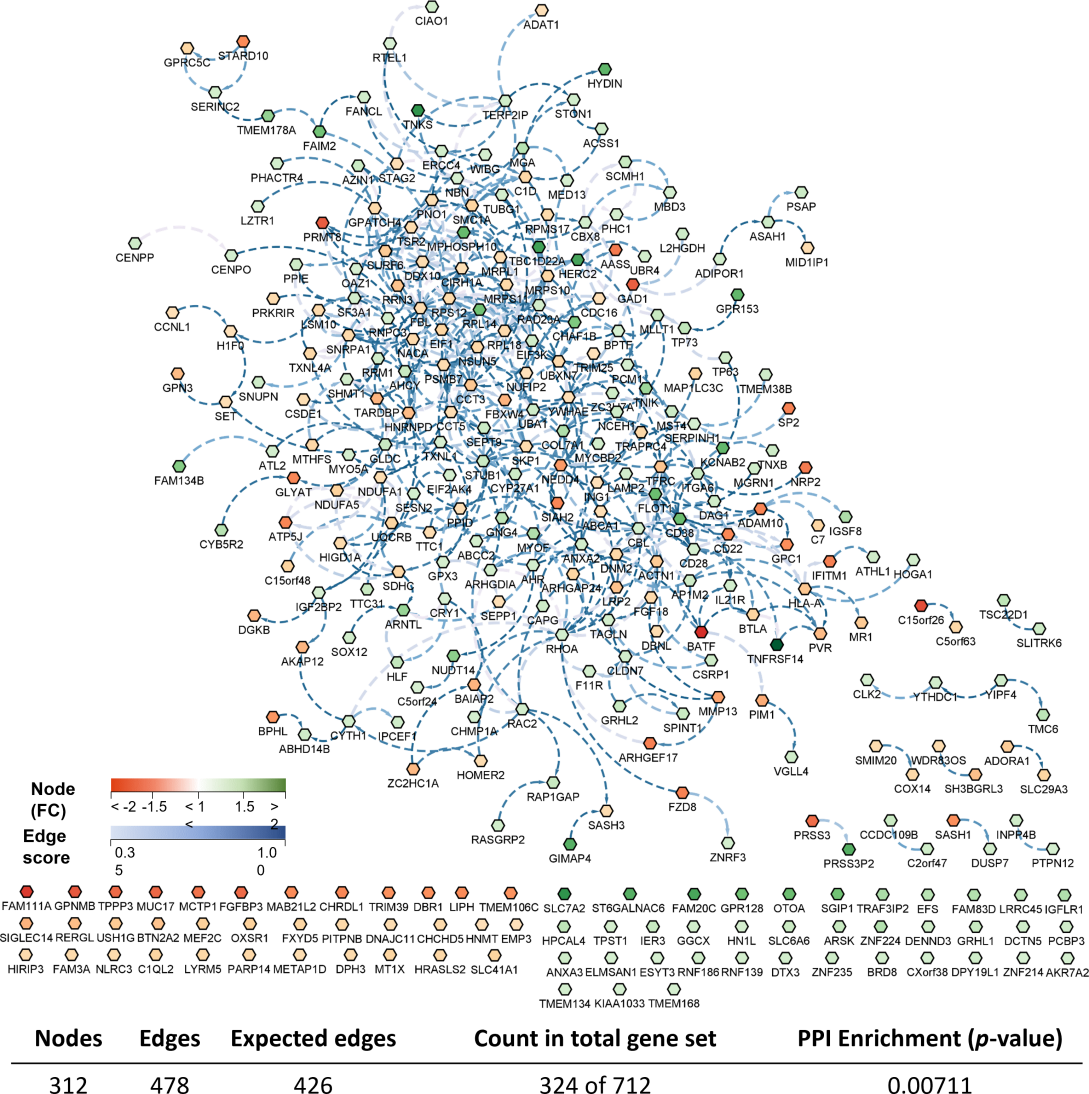
Biological network of the differentially expressed genes (DEGs) in the skin of gilthead seabream (*Sparus aurata*) fed experimental low fishmeal diets containing *Debaryomyces hansenii* (1.1%, 17.2 x 10^5^ cfu; Yeast diet) or devoid of the probiotic (Control diet). Node fills with a continuous mapping color from red (down-regulated DEGs) to green (up-regulated DEGs) represent the fold change (FC) intensity. The edge score was represented by dotted lines with a continuous mapping from 0.3 (light Blue) to 1.00 (dark blue). The transcripteractome was obtained using Cytoscape (v3.9.1) platform with String (v1.7.1). For details, please refer to **Supplementary Table 1**.

Considering the complete list of DEGs, a transcriptomic-based biological network (transcripteractome) was generated containing 312 nodes (DEGs) which resulted in 478 interactions (edges) (**Figure 5**). The remaining DEGs (N = 388) were annotated as unknown genes and, therefore, these were excluded from the current analysis.

#### 3.3.2 Functional enrichment analysis

**Figure 6:**
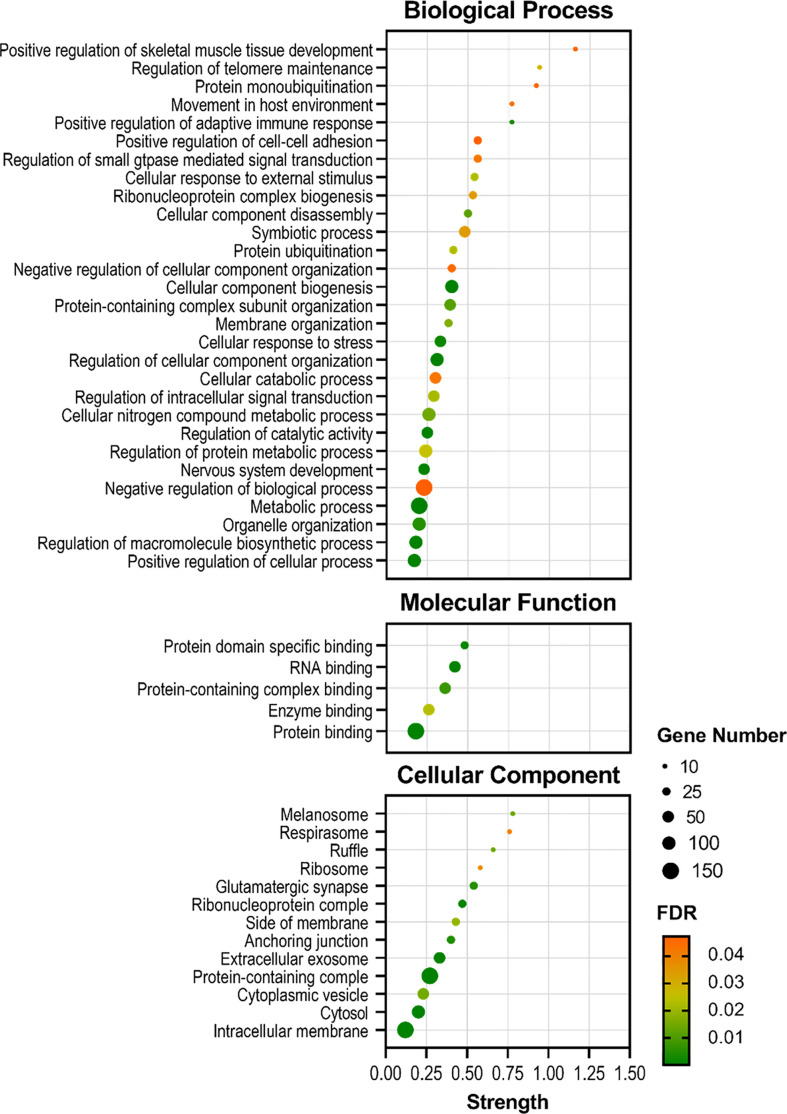
Bubble plots showing the enrichment analysis of the largest DEGs subnetwork (*P* < 0.05) in the skin of gilthead seabream (*Sparus aurata*) fed experimental low fishmeal diets containing *Debaryomyces hansenii* (1.1%, 17.2 x 10^5^ cfu; Yeast_1.1%_ diet) or devoid of the probiotic (Control diet). The axis variable assignments show: x-axis = strength (Log_10_(genes observed/genes expected)); y-axis = GO terms (in Biological Process, Molecular Function, and Cellular Component aspects). The size of the black circles associates with the gene number involved in each GO term. The color palette from green (low) to red (high) denotes the False Discovery Rate (FDR; significance of the enrichment *p*-values) of each GO term. For details, please refer to **Supplementary Table 2**.

The enrichment analysis identified several biological processes and pathways in the skin of *S. aurata* when comparing both experimental groups. Thus, most of the Gene Ontology (GO) annotations obtained from the transcripteractome were associated with metabolic, regulatory, and symbiotic interaction-related processes. Selecting the genes associated with the largest subnetwork, which denotes the greatest quantity of interactions described, the enrichment analysis identified 29 GO Biological Processes (GO-BP), 5 GO Molecular Functions (GO-MF), and 13 GO Cellular Components (GO-CC) (**Figure 6** and **Supplementary Table 2**), increasing the protein-protein interaction enrichment *p*-value up to 1.75 x 10^12^.

**Figure 7:**
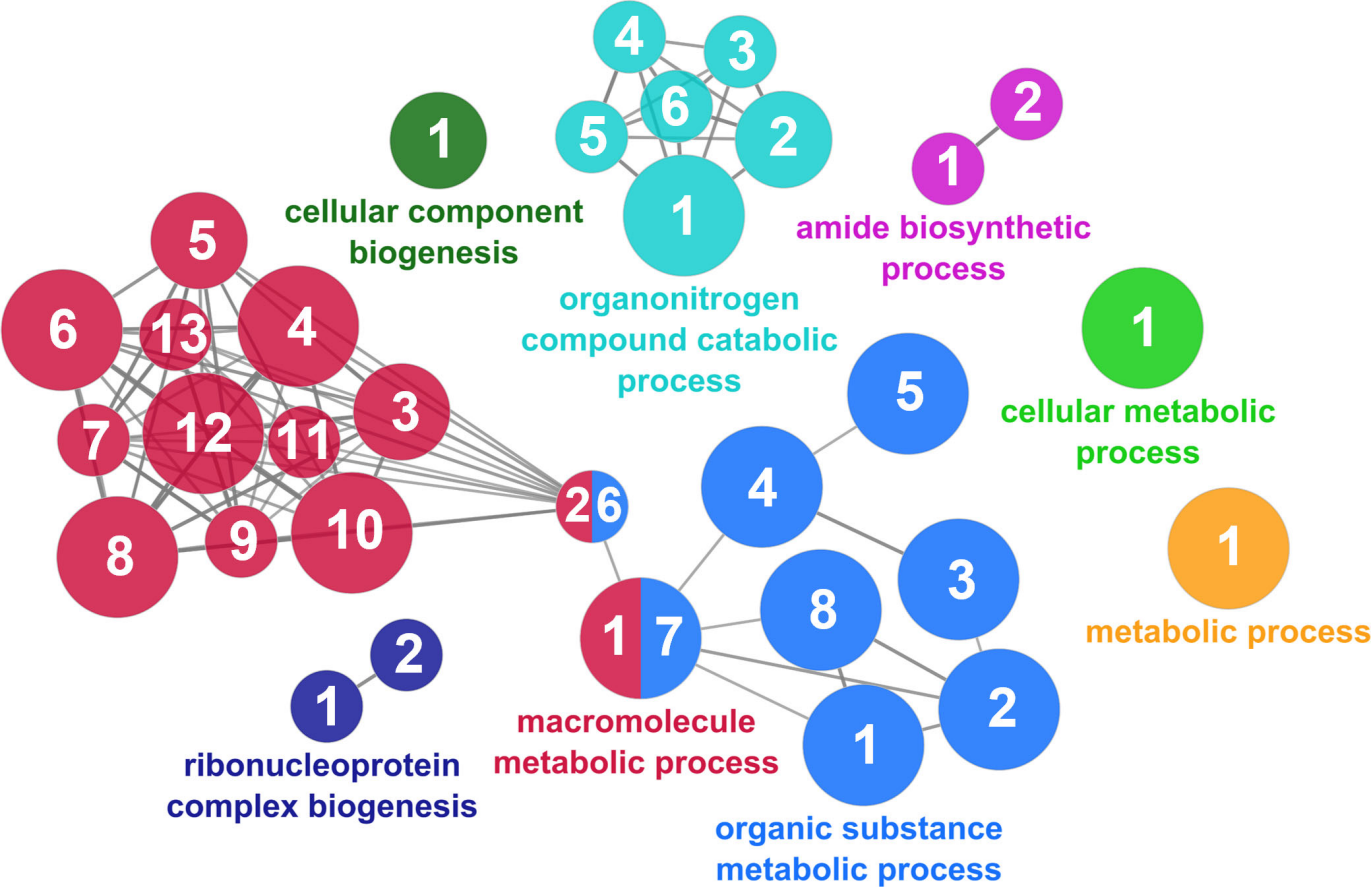
The metabolic cluster of the functional enrichment network for biological processes based on the related functions of the differentially expressed genes (DEGs) in the skin of gilthead seabream (*Sparus aurata*) fed experimental low fishmeal diets containing *Debaryomyces hansenii* (1.1%, 17.2 x 10^5^ cfu; Yeast_1.1%_ diet) or devoid of the probiotic (Control diet). The node size determines the significance (Bonferroni step-down correction; small *p* < 0.05; medium *p* < 0.005; large *p* < 0.0005), the node color determines the principal GO process aggrupation, and the node numbers indicate different GO processes classified in **Supplementary Table 3**.

**Figure 8:**
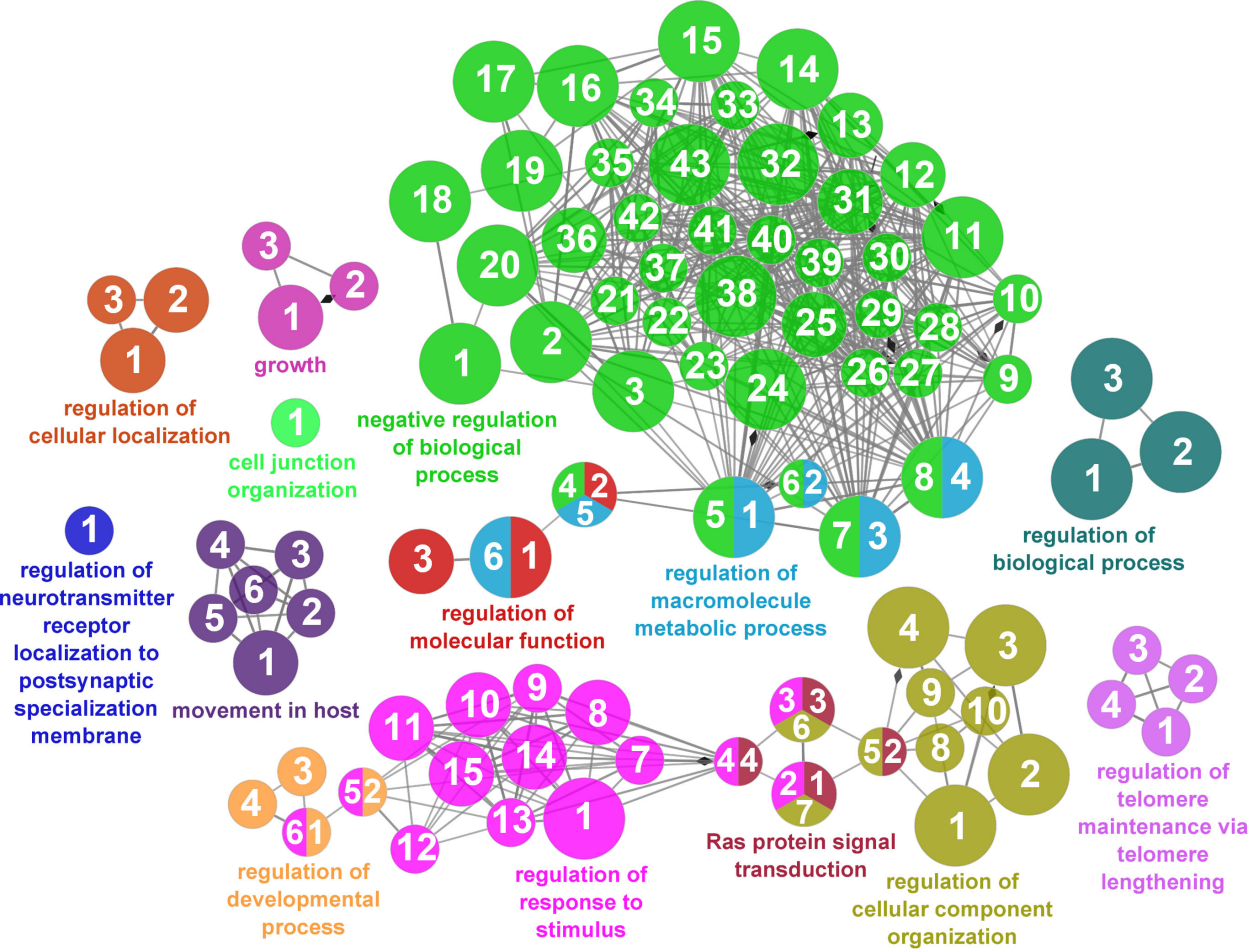
Regulatory mechanisms cluster of the functional enrichment network for biological processes based on the related functions of the differentially expressed genes (DEGs) in the skin of gilthead seabream (Sparus aurata) fed experimental low fishmeal diets containing Debaryomyces hansenii (1.1%, 17.2 x 105 cfu; Yeast1.1% diet) or devoid of the probiotic (Control diet). The node size determines the significance (Bonferroni step-down correction; small p < 0.05; medium p < 0.005; large p < 0.0005), the node color determines the principal GO process aggrupation, and the node numbers indicate different GO processes classified in **Supplementary Table 3**.

**Figure 9:**
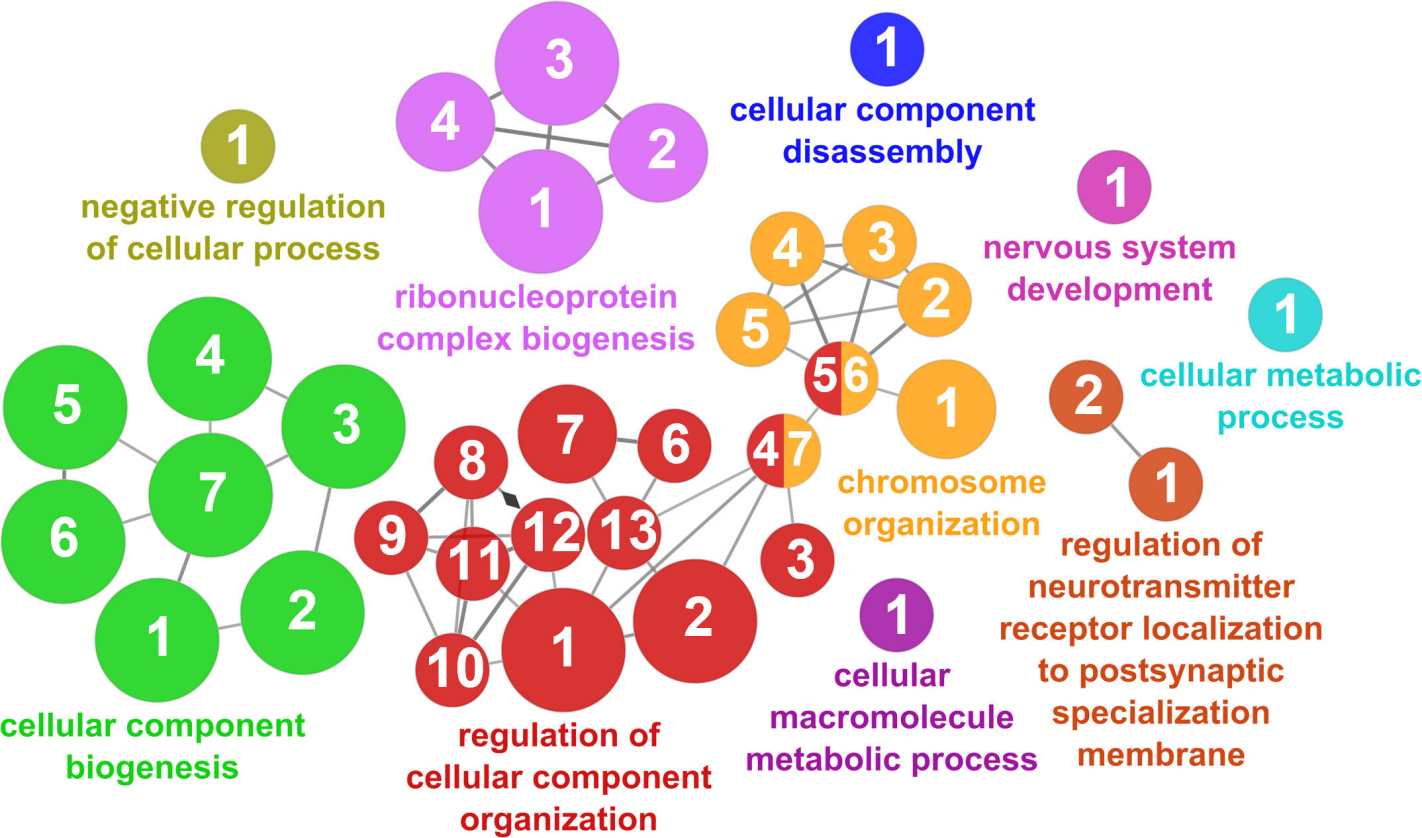
Cellular component organization cluster of the functional enrichment network for biological processes based on the related functions of the differentially expressed genes (DEGs) in the skin of gilthead seabream (*Sparus aurata*) fed experimental low fishmeal diets containing *Debaryomyces hansenii* (1.1%, 17.2 x 10^5^ cfu; Yeast_1.1%_ diet) or devoid of the probiotic (Control diet). The node size determines the significance (Bonferroni step-down correction; small *p* < 0.05; medium *p* < 0.005; large *p* < 0.0005), the node color determines the principal GO process aggrupation, and the node numbers indicate different GO processes classified in **Supplementary Table 3**.

**Figure 10:**
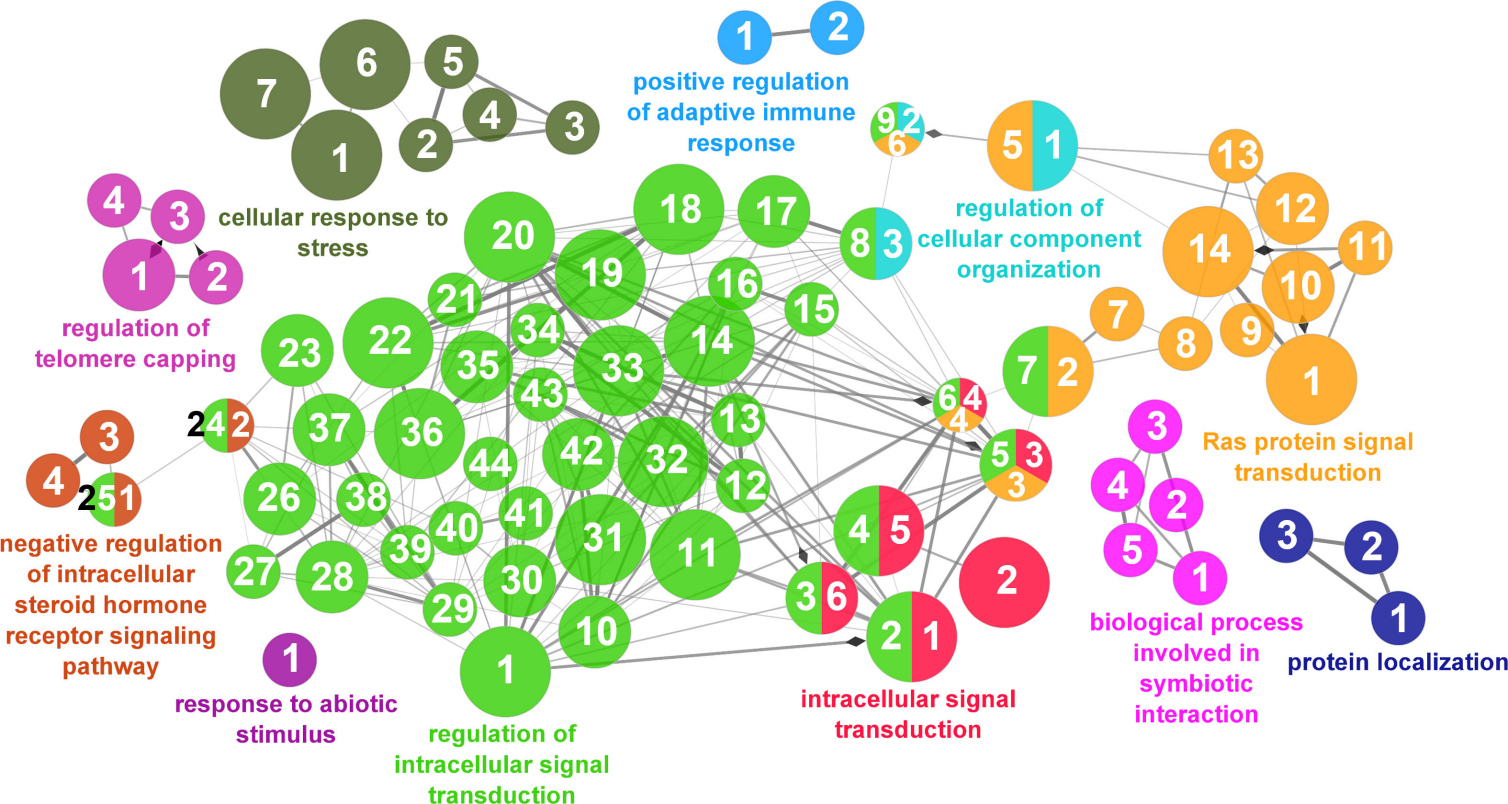
Response to stimulus cluster of the functional enrichment network for biological processes based on the related functions of the differentially expressed genes (DEGs) in the skin of gilthead seabream (*Sparus aurata*) fed experimental low fishmeal diets containing *Debaryomyces hansenii* (1.1%, 17.2 x 10^5^ cfu; Yeast_1.1%_ diet) or devoid of the probiotic (Control diet). The node size determines the significance (Bonferroni step-down correction; small *p* < 0.05; medium *p* < 0.005; large *p* < 0.0005), the node color determines the principal GO process aggrupation, and the node numbers indicate different GO processes classified in **Supplementary Table 3**.

**Figure 11:**
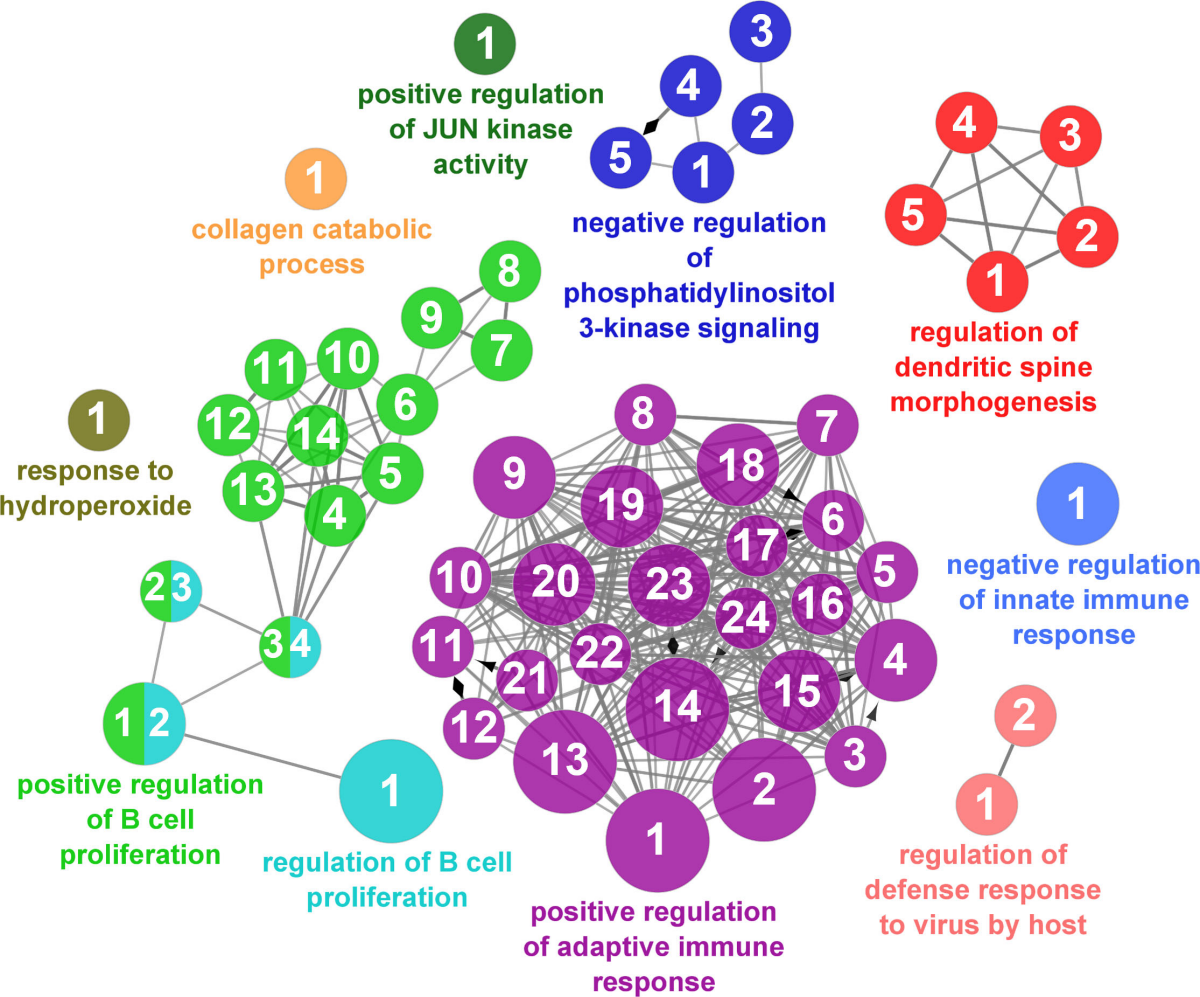
Immune processes cluster of the functional enrichment network for biological processes based on the related functions of the differentially expressed genes (DEGs) in the skin of gilthead seabream (*Sparus aurata*) fed experimental low fishmeal diets containing *Debaryomyces hansenii* (1.1%, 17.2 x 10^5^ cfu; Yeast_1.1%_ diet) or devoid of the probiotic (Control diet). The node size determines the significance (Bonferroni step-down correction; small *p* < 0.05; medium *p* < 0.005; large *p* < 0.0005), the node color determines the principal GO process aggrupation, and the node numbers indicate different GO processes classified in **Supplementary Table 3**.

**Figure 12:**
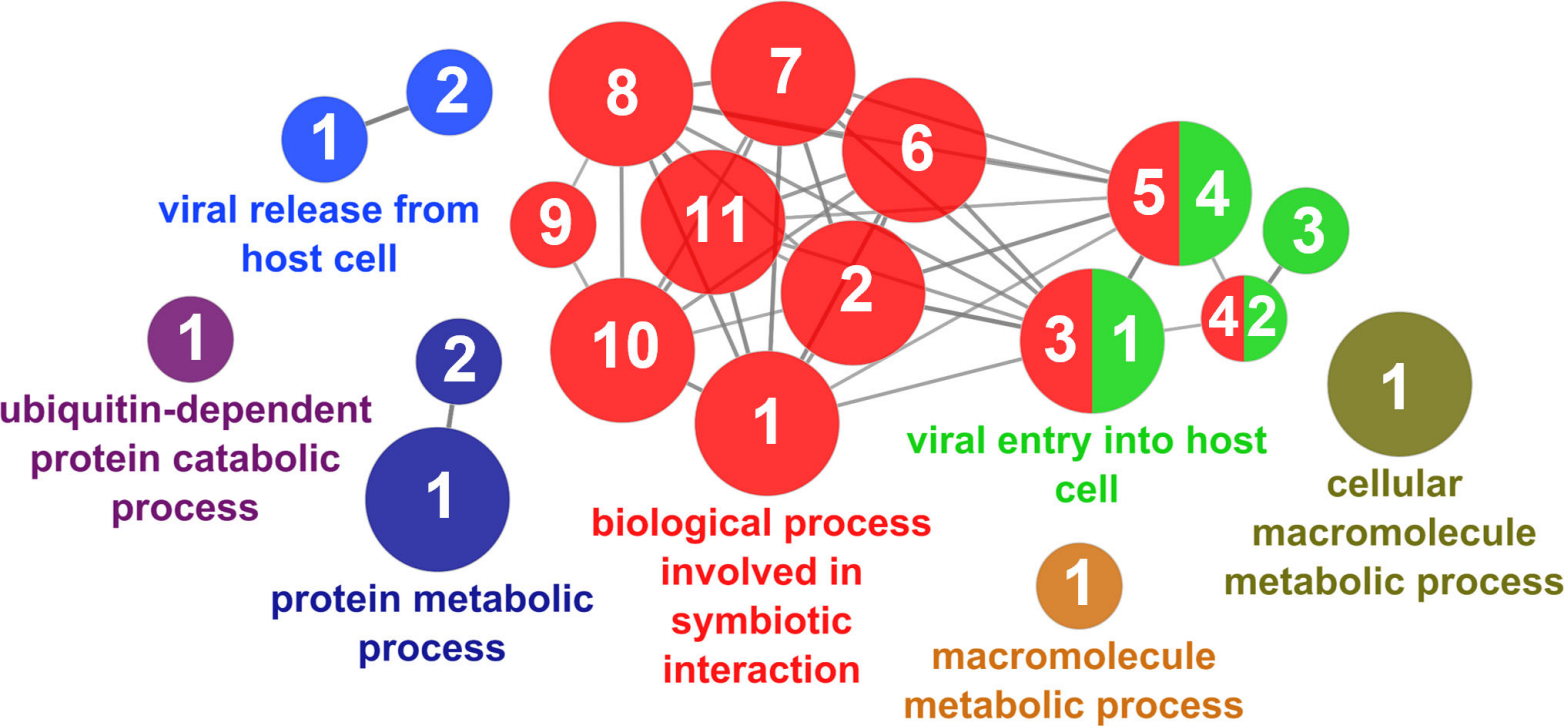
Interspecies interaction between organisms cluster of the functional enrichment network for biological processes based on the related functions of the differentially expressed genes (DEGs) in the skin of gilthead seabream (*Sparus aurata*) fed experimental low fishmeal diets containing *Debaryomyces hansenii* (1.1%, 17.2 x 10^5^ cfu; Yeast_1.1%_ diet) or devoid of the probiotic (Control diet). The node size determines the significance (Bonferroni step-down correction; small *p* < 0.05; medium *p* < 0.005; large *p* < 0.0005), the node color determines the principal GO process aggrupation, and the node numbers indicate different GO processes classified in **Supplementary Table 3**.

**Figure 13:**
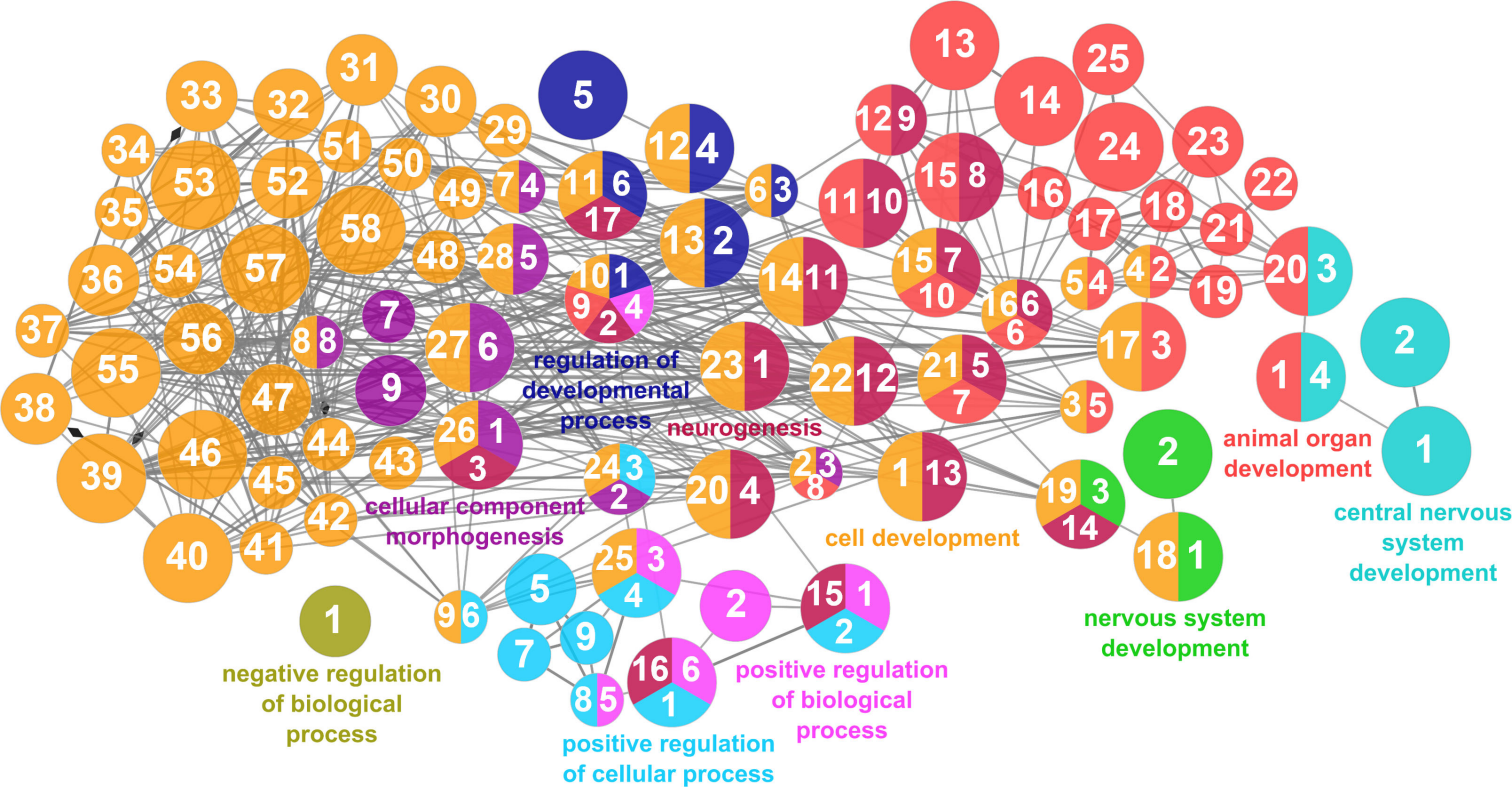
Developmental processes cluster of the functional enrichment network for biological processes based on the related functions of the differentially expressed genes (DEGs) in the skin of gilthead seabream (*Sparus aurata*) fed experimental low fishmeal diets containing *Debaryomyces hansenii* (1.1%, 17.2 x 10^5^ cfu; Yeast_1.1%_ diet) or devoid of the probiotic (Control diet). The node size determines the significance (Bonferroni step-down correction; small *p* < 0.05; medium *p* < 0.005; large *p* < 0.0005), the node color determines the principal GO process aggrupation, and the node numbers indicate different GO processes classified in **Supplementary Table 3**.

According to the results reported by the enrichment analysis, the GOs were classified into seven representative clusters of Biological Processes as depicted in **Figures 7** **–** **13** and **Supplementary Table 3**. The largest cluster was related to metabolic pathways (**Figure 7**) and contained 171 DEGs (54.8% of known DEGs). The second cluster was related to regulatory mechanisms (**Figure 8**) and was represented by 140 DEGs (44.9% of the known DEGs). The third cluster encompassed cellular processes and specifically those involved in the cellular component organization (**Figure 9**), with 104 associated DEGs (33.3% of known DEGs). The following three clusters were related to defense and environmental interactions: a cluster of response to stimulus (72 DEGs; 23.1% of the known DEGs) (**Figure 10**), a cluster related to immunity (61 DEGs; 19.6% of the known DEGs) (**Figure 11**), and a cluster related with biological process involved in interspecies interaction between organisms (29 DEGs; 9.3% of the known DEGs) (**Figure 12**). The last cluster grouped 46 DEGs (14.7% of the known DEGs) and was related to developmental processes (**Figure 13**).

In order to elucidate the main activities of DEGs that occur at the molecular level, an enrichment analysis was performed to elucidate different molecular functions related to binding functional terms (**Figure 6**). Results showed that at the molecular level, the functions of DEGs in fish fed the Yeast_1.1%_ diet were mainly related to protein-containing complex, enzymatic, and RNA-binding associations. Additionally, the locations relative to the cellular structure in which the DEGs performed these biological and molecular functions were mainly structural and at cytosolic spaces. Anchoring, membrane, and vesicle cellular components showed to be the locations of the majority of the DEG products, and to a lesser extent, among some cellular components like the ribosome, respirasome, and melanosome structures. These functions and distributions indicate an increased interaction and response modulations on fish skin cells produced by the dietary supplementation of *D. hansenii*.

## 4 Discussion

The modulation of health and immunity through dietary strategies is considered a reliable alternative to the use of chemicals and drugs, in addition to being considered a proactive and sustainable practice in farmed animals (40). To this end, probiotic, prebiotic, and symbiotic supplements in diets, together with their constitutive bioactive components, offer viable alternatives to the use of chemical modulators, improving health and fish defense in addition to providing benefits in animal performance and/or improving several physiological processes (41, 42). This is of special interest to the aquaculture industry, a sector vulnerable to different biotic and abiotic challenging episodes, which usually works with shorter margins of error compared to other production industries (43, 44). With this aim, the present study explored the effects of *D. hansenii* (CBS 8339) supplementation in a low fish meal-based diet on the skin mucosa of *S. aurata* juveniles. Unlike other mucosal tissues, skin covers the majority of the fish’s body surface. Therefore, it acts as the first frontier between the animal and the surrounding environment, giving it a role as the main defensive barrier.

The holistic approach proposed in this study combined a transcriptomic analysis of the skin tissue and the study of its exuded mucus in terms of SMABs and its bacterial defense capacity. When included in the diet, *D. hansenii* resulted in an improvement of somatic growth and feed efficiency parameters and supported the immune system in *S. aurata*. However, the highest supplementation level tested (2.2%) did not provide any significant improvement among the key performance indicators assessed in the current study (same levels as control), which indicated that there was not a dose-response effect of the probiotic administration, suggesting the 1.1% level as the optimal supplementation (45). Therefore, in this study, the Yeast_1.1%_ diet showed enhanced performance indicators, which agree with other studies reporting the improvement of the fish condition in *S. aurata* at similar levels (46, 47). However, current results differ from other freshwater species presenting no advantages at any supplementation level (48). The significance of these findings was already discussed by Sanahuja et al. (21). In previous reports, it was described that *D. hansenii* possesses bioactive components with immunomodulatory and digestive promotion effects, like β-glucans and polyamines, whose actions are reported to be indispensable for some biochemical pathways related to cell differentiation and proliferation (19, 22, 49). To test if these effects benefit the skin mucosa, the supplementation level was considered at 1.1% since ultimately, to be considered beneficial for animal health and welfare, the improvement of performance is decisive along with its defensive capacity against biotic and environmental agents.

### 4.1 Modulatory effects of dietary *D. hansenii* supplementation

A detailed transcripteractome was performed from Biological processes, Molecular functions, and Cellular components to unravel the modulatory effects of the yeast-based supplemented diet on the skin mucosal tissue. The enrichment analysis revealed multiple GO annotations corresponding to binding molecular functions, mainly “Protein binding” (GO.0005515), “Enzyme binding” (GO:0019899), and “Protein-containing complex binding” (GO:0044877). In addition, these functions are associated with the presence of regulatory biological processes, which might be correlated with the defensive pluripotency attributed to the fish skin. Specifically, among the main mucosal tissues (i.e., gill, gut, and skin), the skin is considered the principal defensive barrier since it covers almost the entire body of the fish (6, 50). In this line, the analysis of the “Cellular component” GO showed that the DEGs performed their functions, especially in membranes (“Intracellular membrane”, GO:0043231; “Side of membrane”, DO:0098552) in addition to being part of vesicles and exosomes (“Cytoplasmic vesicle”, GO:0031410; “Extracellular exosome”, GO:0070062). In fish, the skin tissue is composed of active cells, with a high turnover ratio to maintain the viability and protective barrier function of this tissue (51). Moreover, the skin epidermis also contains specialized mucus-producing cells which generate and secrete the mucus layer that covers the skin. Therefore, the relation of these biological aspects could represent an improvement in the barrier function of the skin, either by modulating the secretion and/or the composition of the mucus (52) or by enhancing the physical protection capacity at cell-cell junctions or/and cell and extracellular matrix levels (“Anchoring junction”, GO:0070161). These effects are crucial to protecting the organism from environmental fluctuations and biotic stressors (5, 53). The above-mentioned structural and functional characteristics based on the transcriptomic data obtained demonstrate that the supplementation at 1.1% of *D. hansenii* in a low fish meal-based diet (7%) was capable of modulating and supporting skin functions in *S. aurata*. These results may be attributed to the contribution of bioactive compounds from *D. hansenii* since this yeast species is rich in β-glucans, polyamines, and mannan oligosaccharides (19, 20, 54).

Different studies in fish have shown the possibility of modulating skin functions through dietary strategies (27, 30). In our study, we have observed a modulation of some pathways related to amide metabolism (*aass, abcc2, acss1, adam10, asah1, eif1, eif3k, glyat, mrpl1, mrps10, mrps11, mthfs, naca, psap, rpl14, rpl18, rpms17, rps12*, and *shmt1*) and amino acid metabolism (*shmt1, gldc, glyat, aass, ahcy, serinc2*, and *arhgef17*). Similar findings related to the modulation of amide metabolism were reported in a parallel study in which we evaluated the effect of *D. hansenii* dietary supplementation at the intestinal level in the same fish species (21); the results may be attributed to the high content of polyamines in this yeast (19). Polyamines are a part of and regulate a large variety of key cellular processes, such as cell growth, differentiation, or proliferation, whose control is crucial to maintain cell homeostasis (55). In the skin, we found two upregulated genes involved in polyamine regulation, *oaz1* and *azin1*. In response to elevated levels of polyamines, ornithine decarboxylase antizyme 1 (OAZ1) and antizyme inhibitor 1 (AZIN1) regulate the entry of polyamine compounds into cells and may inhibit ornithine decarboxylase (ODC). Thereby, these molecules initially regulate polyamine metabolism (55, 56). In addition to this pathway, the *slc6a6* gene was upregulated in our study, whose function is the transport of taurine, urea, and biogenic amine (57). This modulation of amide metabolism and its related transport mechanisms are undoubtedly of great interest since *D. hansenii* dietary supplementation not only has effects at the intestinal level (21) but also at the skin mucosa level as herein observed, which may indicate a similar effect at cell growth, differentiation, or proliferation levels in different MALTs. Moreover, polyamines and their derived compounds participate in protein metabolism, which is highly relevant in carnivorous fish (58). In this sense, the enrichment analysis revealed several biological processes involved in the regulation of protein turnover, suggesting a modulation of the amino acid and protein metabolism in the fish skin by *D. hansenii* supplementation. Our study also revealed that the *slc7a2* gene, an amino acid transporter member of the amino acid-polyamine-organocation (57, 59), was upregulated in the skin cells. The SLC7A2 transporter is responsible for the cellular uptake of arginine, lysine, and ornithine. An increased uptake of arginine and ornithine, which also participates in polyamine metabolism, supports the above-mentioned hypothesis of increased polyamine functions in the skin through dietary uptake. Moreover, the expression and functions of SLC7A2 have been highly related to both innate and adaptive immunity. In particular, SLC7A2 has been reported to control macrophages’ function toward a bacterial infection and under inflammation in mammal peripheral monocytes (60). The up-regulation of these DEGs related to protein metabolism and polyamine turnover might explain a more efficient use of protein sources (61), which would support the improvement of intestinal health status and nutrient digestion (21), altogether with modulation of immune-related processes.

Furthermore, several mediators of the immune response were also observed to be upregulated in the skin of *S. aurata* fed the Yeast_1.1%_ diet. For instance, the TNF Receptor Superfamily Member 14 (*tnfrsf14*) gene expression was highly increased. The TNFRSF14 membrane-bound receptor binds several ligands mediating both coinhibitory and costimulatory pathways. Therefore, TNFRSF14 regulates immune cell homeostasis (62–64). In accordance, among its ligands, we found that it down-regulated the B- and T-lymphocyte attenuator (*btla*) gene, a member of the CD28 family, and its interaction with TNFRSF14 delivers a coinhibitory signal regulating T-cell activation (65). However, due to their wide cellular distribution, their functional analysis is complex. For instance, the balance herein observed in their expressions suggests a naïve or final stage of the T-cell activation phase, and/or an immature phase of dendritic cells (66). In this line, we observed the upregulation of the *cd28* gene, a costimulatory receptor that triggers T-cell activating functions. Thus, the lack of modulation of costimulatory T cell receptors (TCR), like their cognate CD80/CD86 ligands, and/or interleukin modulatory expressions, allowed us to discard a proliferative T-cell response associated with the upregulation of CD28, supporting the idea of a naïve/resting stage of T-cells modulation in the skin of *S. aurata* after feeding with the Yeast_1.1%_ diet for 70 days (67, 68). In addition, T-cell activation requires, among the above-mentioned signals, a complex formed by MCH with T-cell receptors (TCR) (69). Under this scenario, the downregulation of the human leukocyte antigen (*hla-a*) gene, a major histocompatibility complex class I (MHC-I) molecule present in the cell surface of all nucleated cells, along with genes related to “positive regulation of T cell activation” (*btla, tfrc*, and *sash3*; GO:0050870), coupled to the modulation of genes related to “regulation of T cell proliferation” (*sox12*, and *btn2a2*; GO:0042129), would also support the above-mentioned hypothesis about the T-cell stage found under the current experimental conditions. Regarding B-cell regulation, one of the co-stimulatory signals for B-cell activation is the CD22 molecule as it has been described in turbot (*Scophthalmus maximus*) and Japanese flounder (*Paralichthys olivaceus*) (70). In our study, fish fed the Yeast_1.1%_ diet presented a downregulation of the *cd22* gene, which has been suggested as one of the B-cell biomarkers in Atlantic salmon (*Salmo salar*) as it is consistently expressed in IgM^+^ or IgT^+^ lymphoid cells together with *cd79a* (71). Under the current experimental conditions, the enrichment analysis of DEGs showed several regulatory processes related to B-cell proliferation and adaptative immune responses coupled with the observed upregulation of the *cd38* gene. In this sense, CD38 participates in the regulation of intracellular Ca^2+^, a cell signaling system, which in turn modulates immune functions. In fish, CD38 has been shown to participate in antiviral and antibacterial B-cell responses, and its expression seems to modulate different B-cell stages (72). However, the enrichment analysis herein shows several repressed genes involved in the positive activation and proliferation of B-cells (i.e., *mef2c, tfrc*, and *sash3*; GO:0050871 and GO:0030890, respectively). The functionality and modulation of these above-mentioned lymphocyte-related genes reinforced the hypothesis of an immunomodulatory effect of *D. hansenii* on the skin of fish. With the downregulation of the membrane-attack complex member *c7* gene, its function and expression have been observed in response to infection in fish (73), and the modulation of several viral processes and antiviral genes (i.e., *trim25, pvr, f11r*, among others) suggest that the supplementation of *D. hansenii* in low fish meal-based diets is capable of modulate and regulate immune functions in the skin of *S. aurata*. Similar results related to T and B cell development were reported when comparing the effect of *D. hansenii* on the skin (present study) and the gut in *S. aurata* fed the Yeast_1.1%_ (21) even though different DEGs were involved in this MALTs, which may be attributed to different tissue-specific response to dietary immunomodulators, being that the gut is more tolerogenic than the skin (3, 50).

Regarding the function of the skin protecting the fish, one of the most distinctive features is the production and secretion of mucus by goblet cells and club cells (74). The mucus layer is mainly composed of glycoprotein molecules called mucins, soluble proteins, several metabolites, and hormones, among other compounds (28, 34). In our study, we found that *D. hansenii* dietary supplementation could modulate mucin properties through mucin synthesis and secretion. For example, through the upregulation of the GalNAc sialyltransferase (*st6galnac6*) gene, which adds terminal sialic acids contributing to the mucin negative charges (75). This is of special relevance since sialic acid can act as ligands for receptors on viruses, acting as competitive inhibitors, blocking receptor binding, and limiting infection of epithelial cells (76, 77). Moreover, the upregulation of the *myo5a* gene, a class V myosin motor capable of moving along actin filaments (78), and the *esyt3* gene, a synaptotagmin Ca^2+^ sensor required for vesicle fusion, might suggest an increased mucin secretion (75, 79). However, this hypothesis could not be supported by an increase in the expression of MUC18, which has been described as the most abundant mucin in *S. aurata* that plays an important role in adhesion and protection against pathogens (80). In our study, we found a repressed expression of *muc17*, a membrane-bounded and no secreted mucin, which could indicate a differential modulatory pattern for skin mucins under the present experimental conditions. However, the available information regarding this specific issue is limited, and further studies are needed to discern the complex pattern of mucins, their changes, and their implications in skin mucus homeostasis.

Regarding mucus composition, the SMABs profile analyzed did not show major changes except for a slight increase in skin mucus glucose levels. Glucose, lactate, and protein levels are markers of metabolic and stress factors in fish mucus (34). Increased glucose levels could indicate a stressful condition or a switch in metabolic status. However, the levels of cortisol, the major corticosteroid indicator of stress in fish (81), remained constant through dietary groups. Indeed, when the levels of metabolites were standardized per protein ratios, the Control and Yeast_1.1%_ diets did not show differences in glucose levels, but higher levels on the Yeast_2.2%_ were exhibited. Taking this into consideration, these results also supported that the above-mentioned hypothesis of increased mucus production, as transcriptomic data hinted, is due to the inclusion of yeast in diets. As increased mucus production is shown to be the first defensive mechanism in fish (82), this might be considered a beneficial measure against potential biotic and abiotic stressors due to its incorporated defensive components, as the *in vitro* skin mucus co-culture with pathogenic bacteria also indicated. Among the above-mentioned metabolites, the skin mucus is also composed of a great variety of innate immune components and defensive molecules that, working together, confer the defensive capacity to the mucus (5, 83). Hernández-Contreras et al. (31) found increased activity of several immune-related enzymatic parameters in the skin mucus (e.g., proteases and lysozyme) when fish were fed a diet supplemented with *D. hansenii*. In our study, some enzymatic-related genes were modulated, specifically two protease genes (*prss2*, and *prss3*), and one phosphatase inhibitor-related gene (*set*). The upregulation of the serine protease 2 (*prss2*), which participates in the formation of antimicrobial peptides (AMP) (84, 85), could suggest an increased AMPs level, potentiating the antibacterial defense. The modulation of these defense components has been shown in the grater amberjack fish (*Seriola dumerili*) when fed a diet supplemented with yeast-derived mannan oligosaccharides (54). This suggests that yeast components might modulate skin defense components potentiating its defensive capacity. Accordingly, the observed reduced growth capacity of *V. anguillarum* in the skin mucus of *S. aurata* fed yeast-supplemented diet is especially relevant because *Vibrio* strains are capable to adhere to surface cells and penetrate host epithelial and vascular tissues (86). Besides, *P. anguilliseptica* growth was also affected, exhibiting in the final 2 hours of co-culture a decreased growth. *Pseudomonas* is considered an opportunistic pathogen whose infections in *S. aurata* occur under environmental stress (87). The results observed herein suggest that feeding the fish with *D. hansenii* not only could increase mucus production but also could increase its enzymatic and antibacterial defense components, thus protecting the host from potential pathogenic bacteria (88).

In the same line, we found the upregulation of antioxidant-related DEGs (*txnl1, gpx3, ipcef1*, and *sesn2*). The antioxidant modulatory defense effects of *D. hansenii* have been demonstrated in different animal species including fish (46, 89, 90). However, this modulating effect is not reflected in the total antioxidant capacity of the skin mucus, whose values in the Yeast_1.1%_ diet remained close to those from the Control diet. Under certain situations, the skin mucus’s antioxidant capacity is promoted to protect the mucosal surface from oxidative attack, either due to environmental stress (91, 92) or skin disorders (93). Due to the lack of oxidative challenging situations, we hypothesize that the dietary yeast supplementation promoted sentinel antioxidant defensive processes rather than activating response stress by reactive oxygen species (ROS) presence, which is in agreement with other studies focused on functional feeds (30, 32). In addition, skin transcriptomics did not reflect responses linked to oxidative damage but to the downregulation of several genes involved in oxidative phosphorylation, which is the main metabolic ROS producer in cell metabolism (94, 95). This may support the hypotheses that *D. hansenii* supplementation might result in a better condition of the skin, including a tight regulation of its immune response, enabling a more efficient use of available energy and nutrients for growth (21).

## 5 Conclusions

The present holistic study of the skin response to the dietary administration of the probiotic *D. hansenii* in low fish meal-based diets when administered to *S. aurata* juveniles provides new insights into its effects on fish skin condition and defensive function. The results observed herein on fish performance indicators demonstrated that *D. hansenii*, when supplemented at 1.1% (17.2 x 10^5^ cfu), enhances *S. aurata* juveniles’ growth performance and feed efficiency, whereas when supplemented at higher levels (2.2%; 35.7 x 10^5^ cfu) do not provide any advantages. In addition, at the transcriptomic level, *D. hansenii* supplementation modulated innate immunity (i.e., T and B cell differentiation), increasing skin barrier and cell trafficking functions, potentiating the physical barrier of this tissue while maintaining its homeostatic immune and metabolic status. Besides, the skin mucus’s defensive capacity was enhanced by increasing its protective function against bacterial fish pathogens, which was associated with immunomodulatory compounds found in yeast. Thus, these results support the dietary administration of *D. hansenii* as an excellent tool to promote the skin mucosa defensive capacity, which is the first line of fish defense against environmental and biotic challenges, when included in low fish meal-based diets for *S. aurata*.

## Data availability statement

The datasets presented in this study can be found in online repositories. The names of the repository/repositories and accession number(s) can be found below: https://www.ncbi.nlm.nih.gov/, GPL13442, https://www.ncbi.nlm.nih.gov/, GSE162504.

## Ethics statement

The animal study was approved by This study was conducted following the Guiding Principles for Biomedical Research Involving Animals (EU2010/63), the guidelines of the Spanish laws (law 32/2007 and RD 53/2013) and authorized by the Ethical Committee of the Institute for Research and Technology in Food and Agriculture (Spain), and Generalitat de Catalunya government for the use of laboratory animals (FUE-2020–01719167). The study was conducted in accordance with the local legislation and institutional requirements.

## Author contributions

EG designed and carried out the experiments. Biological samplings were performed by EG, LF-A, JF, AI, and IS. The transcriptomic data analysis and interpretation were performed by IS, ST, EV-V, and FR-L. LF-A and IS carried out the skin mucus analyses. DT-R reviewed and validated the methodology used in the study. The study was supervised by EG, AI, LT, and IS. IS wrote the original draft. All the authors provided critical feedback, read, and agreed to the published version of the manuscript.
